# Relationship Between Circulating Netrin-1 Concentration, Impaired Fasting Glucose, and Newly Diagnosed Type 2 Diabetes

**DOI:** 10.3389/fendo.2018.00691

**Published:** 2018-11-23

**Authors:** Jisook Yim, Gyuri Kim, Byung-Wan Lee, Eun Seok Kang, Bong-Soo Cha, Jeong-Ho Kim, Jin Won Cho, Sang-Guk Lee, Yong-ho Lee

**Affiliations:** ^1^Department of Laboratory Medicine, Yonsei University College of Medicine, Seoul, South Korea; ^2^Division of Special Chemistry, Green Cross Reference Laboratory, Yongin-si, South Korea; ^3^Department of Laboratory Medicine, Veterans General Hospital, Incheon, South Korea; ^4^Division of Endocrinology and Metabolism, Department of Medicine, Samsung Medical Center, Sungkyunkwan University School of Medicine, Seoul, South Korea; ^5^Department of Internal Medicine, Yonsei University College of Medicine, Seoul, South Korea; ^6^Institute of Endocrine Research, Yonsei University College of Medicine, Seoul, South Korea; ^7^Department of Systems Biology, Glycosylation Network Research Center, Yonsei University, Seoul, South Korea

**Keywords:** Netrin-1, type 2 diabetes, impaired fasting glucose, inflammation, relationship

## Abstract

**Background:** The protein netrin-1 has demonstrated anti-inflammatory, tissue regeneration, and immune modulation properties. Although inflammation is a major contributing factor in the development of insulin resistance and type 2 diabetes, little is known about a possible relationship between serum netrin-1 and type 2 diabetes. Therefore, we investigated the association between circulating levels of netrin-1 and glycometabolic parameters predictive of type 2 diabetes.

**Methods:** Serum samples were collected from 41 normal controls, 85 subjects with impaired fasting glucose (IFG), and 92 subjects with newly diagnosed type 2 diabetes. Clinical and laboratory parameters were assessed and netrin-1 levels were measured by commercial enzyme-linked immunosorbent assay. Spearman correlation analyses and multivariable-adjusted regression analyses were conducted to examine the relationship between serum netrin-1 levels and glycometabolic parameters.

**Results:** Serum netrin-1 levels in subjects with type 2 diabetes or IFG were significantly higher compared to normal controls (441.0, 436.6, and 275.9 pg/mL, respectively; *P* for trend < 0.001). Serum netrin-1 levels were significantly positively correlated with fasting glucose, HbA_1c_, and insulin resistance index (all *P*s < 0.01). Serum netrin-1 levels were independently associated with IFG or type 2 diabetes (standardized β = 0.405, *P* < 0.001) after adjusting for covariates and potential confounders. In addition, the receiver operating characteristic (ROC) analysis showed that serum netrin-1 levels could identify the presence of IFG and type 2 diabetes with the area under the ROC curve (AUC) of 0.784 (*P* < 0.001).

**Conclusions:** Our results suggest that elevated serum netrin-1 levels are significantly associated with the presence of IFG and type 2 diabetes.

## Background

As incidence rates of obesity and insulin resistance increases worldwide, the prevalence of diabetes continues to rise dramatically (1). The global population of type 2 diabetes mellitus is expected to grow to more than 366 million people by 2030, from 171 million in 2000 (2). Acute and chronic complications of diabetes and its associated high morbidity produce overwhelming burdens on healthcare systems and society (3). In addition to patients with diabetes, people with impaired fasting glucose (IFG) account for increasing socioeconomic costs associated with substantially increased risks of vascular disease and overall mortality (4, 5). Moreover, undiagnosed diabetes in young people is associated with an increased risk of cardiovascular disease (6, 7). Early diagnosis and management of type 2 diabetes is of critical importance and should be emphasized due to the often silent symptoms of its progression. Thus, reliable biomarkers to identify IFG or type 2 diabetes are needed.

Netrin-1, which belongs to a family of laminin-related proteins, has been reported as a neuronal guidance cue, acting as both a chemo-attractant and as a chemo-repulsive force during axonal migration (8, 9). Netrin-1 is primarily expressed in the central nervous system (CNS), but also in non-neural tissues such as vascular endothelial cells, pancreas, liver, spleen, lung, intestine, and kidney (10). In recent studies, netrin-1 was shown to play various other key roles beyond axonal guidance during nervous system development, including organogenesis of mammary glands, lungs, and pancreas, and angiogenesis and tumorigenesis (11–13). In addition, netrin-1 was reported to be involved in leukocyte migration in peripheral organs, in tissue regeneration, and in modulation of inflammation-based conditions (10, 14–18). Moreover, netrin-1 exhibited an anti-angiogenic effect, allowing an improvement in blood flow to hypoxic tissue, and showed a promising cardioprotective capacity in the prevention of ischemia-reperfusion injury by consequent nitric oxide production in animal studies (19–22). Inflammation is a major contributor to the development of diabetes (23). Increased oxidative stress and various pro-inflammatory cytokines and chemokines, including tumor necrosis factor-α (TNF-α) and interleukin-6 (IL-6), which might interfere with anti-inflammatory effects on insulin action, were associated with insulin resistance, obesity, and type 2 diabetes (23, 24). Moreover, there is evidence to suggest that prior to its onset, diabetes displays features of inflammation (25). This notion, coupled with the tissue regenerative and immunomodulatory properties of netrin-1, led us to explore whether circulating levels might be related to the development of IFG or type 2 diabetes. Therefore, we investigated circulating levels of netrin-1 and its associations with clinical parameters in three population groups: normal controls and individuals with IFG and type 2 diabetes.

## Methods

### Study population

Participants were individuals who visited the Diabetes Center at the tertiary-level, university-affiliated Severance Hospital, Seoul, Korea from September 2015 to January 2017. Subjects with current renal or hepatic disease, malignancy, diabetes other than type 2 diabetes mellitus, any acute inflammation or infection, current significant cardiovascular disease, and those taking anti-diabetes medication were excluded from participation. We enrolled the drug-naïve patients with type 2 diabetes and laboratory parameters were determined at diagnosis, considering the effect of anti-diabetes drugs on circulating netrin-1 levels. A total of 218 individuals were enrolled and classified as normal controls (*n* = 41), subjects with IFG (*n* = 85), and subjects with newly-diagnosed type 2 diabetes (*n* = 92). Written informed consent, compatible with the Helsinki Declaration, was provided by participants prior to enrolling into the study, which was approved by the Institutional Review Board of Severance Hospital (IRB No. 4-2015-0503).

### Definition of normal, impaired fasting glucose, and type 2 diabetes

In this study, we defined normal controls as a fasting glucose level < 100 mg/dL and HbA_1c_ < 5.7%), IFG as a fasting glucose level of 100–125 mg/dL or HbA_1c_ of 5.7–6.5% without taking anti-diabetes medication, and newly diagnosed type 2 diabetes as a fasting glucose level ≥126 mg/dL or 2-h postprandial glucose level ≥200 mg/dL or HbA_1c_ ≥6.5% without taking anti-diabetes medication (26). Levels of postprandial glucose were measured in 17.1% (*n* = 7), 68.2% (*n* = 58), and 88.0% (*n* = 81) in individuals with normal, IFG, and type 2 diabetes, respectively.

### Measurement of clinical and laboratory parameters

Personal medical history, smoking status, alcohol consumption habits, and anthropometric data were collected. Subjects were categorized with regard to smoking status and alcohol consumption (non-current or current). Body weight and height were measured, and body mass index (BMI) was calculated as kg/m^2^. Obesity was defined according to the criteria for the Asian and Pacific regions (BMI ≥ 25 kg/m^2^) (27). Blood pressure was measured by using a mercury sphygmomanometer in a sitting positing after at least 5 min of rest. Participants' blood samples were collected after fasting for 8–12 h. Serum samples were placed into Eppendorf cryotubes and stored at −80°C until runtime. HbA_1c_ was determined using the Cobas Integra 800 system (Roche Diagnostics, Germany). Serum glucose, 2-h postprandial glucose, alanine aminotransferase (ALT), aspartate aminotransferase (AST), total cholesterol, triglycerides, and high density lipoprotein (HDL) cholesterol were determined using a Hitachi 7600 analyzer (Hitachi Co., Tokyo, Japan). Fasting serum insulin and C-peptide levels were determined using electrochemiluminescent assay and Cobas e601 analyzer (Roche Diagnostics). Low density lipoprotein (LDL) cholesterol was calculated using the Friedewald equation (LDL cholesterol [mg/dL] = total cholesterol [mg/dL] – HDL cholesterol [mg/dL] – triglycerides [mg/dL]/5). Homeostasis Model Assessment of Insulin Resistance (HOMA-IR) was calculated from the following formula: [fasting plasma insulin (μIU/mL) × fasting plasma glucose (mg/dL) /405] (28, 29). Estimated glomerular filtration rate (eGFR) was calculated based on the CKD Epidemiology Collaboration (CKD-EPI) equation (30). Levels of netrin-1 were measured by commercial enzyme-linked immunosorbent assay (ELISA; Cloud-Clone Corp., Houston, TX, USA) according to the manufacturer's instructions. Three ELISA kits with same lot number (No. L170220861) were used. Each ELISA assay included sera from all the three categories of individuals examined. Although there was no internal control materials, all tested ELISA kits were same lot number and the assays were performed by one researcher on the same day. In addition, the mean netrin-1 concentration of each category of individuals were not significantly different among three ELISA assays supporting that each ELISA assay had comparable results. A total of five calibrators were used except blank, and the blank OD (optical density) value was subtracted from each OD value, and then concentration was calculated by calibration curve. The lowest concentration the method could determine was 52 pg/mL. The coefficient of variation for netrin-1 was < 10% and < 12% in the intra-assay and inter-assay, respectively. The details of the ELISA assay data are available in the Supplementary Table S1.

### Statistical analysis

All continuous variables are presented as means ± standard deviations (SDs), and categorical variables are expressed as *n* (%). Parametric differences were compared using analysis of variance (ANOVA) tests for continuous variables and chi-square tests for categorical variables and, as appropriate, by *post-hoc* Bonferroni tests. To examine the relationship between serum netrin-1 levels and clinical and laboratory parameters, Spearman correlation analyses were conducted. For multivariable-adjusted regression analyses, model 1 was adjusted for age, sex, and BMI; model 2 was further adjusted for HbA_1c_, ALT, HDL cholesterol, eGFR, and the use of statins; and model 3 was further adjusted for HOMA-IR. Variables including HbA_1c_, ALT, HDL cholesterol, eGFR, and HOMA-IR were log-transformed in the multivariable-adjusted regression due to the skewed value distributions. All covariates in the multivariate models had a variance inflation factor (VIF) < 5.0, which was considered adequate to avoid relevant multicollinearity. In addition, to evaluate the viability of serum netrin-1 levels to predict IFG or type 2 diabetes, receiver-operating characteristic (ROC) curves and areas under the ROC curves (AUCs) were determined. A *P* value < 0.05 was considered statistically significant. Statistical analyses were performed using SPSS version 20.0 for Windows (SPSS Inc., Chicago, IL).

## Results

### Baseline characteristics of the study population

Baseline characteristics of study subjects according to glycemic status are shown in Table 1. Among a total of 218 subjects (41 normal controls, 85 subjects with IFG, and 92 subjects with type 2 diabetes), the mean age was 51.7 ± 13.4 years, 51.7% were women, and the mean BMI was 25.2 ± 3.7 kg/m^2^. Compared with normal controls, subjects with IFG or type 2 diabetes were more likely to be male, obese, and to consume more alcohol. Fasting and 2-h postprandial glucose and insulin, HbA_1c_, HOMA-IR, triglycerides, ALT and C-reactive protein (CRP) concentrations were significantly increased in subjects with IFG or type 2 diabetes compared to normal subjects (all *P*s < 0.05). In contrast, the levels of HDL cholesterol were markedly lower in individuals with IFG or type 2 diabetes, compared to normal subjects (*P* < 0.005).

**Table 1:** Characteristics of the study subjects (*n* = 218).

**Variables**	**Normal (*n* = 41)**	**IFG (*n* = 85)**	**Type 2 diabetes (*n* = 92)**	***P-*value**
Age (years)	40.9 ± 14.5^***^	56.0 ± 9.6	52.6 ± 13.4^###^	< 0.001
Female sex	24 (58.5)	49 (57.6)	40 (43.5)	0.107
Current alcohol drinker	21 (4.9)	22 (25.9)	35 (38.0)	< 0.001
Current smoker	1 (2.4)	10 (11.8)	13 (14.11)	0.133
SBP (mmHg)	124.2 ± 16.8	125.0 ± 12.7	127.6 ± 13.1	0.407
DBP (mmHg)	75.4 ± 10.7	76.7 ± 10.4	79.2 ± 11.2	0.281
BMI (kg/m^2^)	22.6 ± 3.3^**^	25.1 ± 3.5	26.3 ± 3.5^###^	< 0.001
Statin use	5 (12.2)	18 (21.2)	23 (25.0)	0.247
Fasting glucose (mg/dL)	90.5 ± 6.2	108.6 ± 7.8^∧∧∧^	169.7 ± 63.2^###^	< 0.001
2-h postprandial glucose (mg/dL)^§^	119.1 ± 23.9	138.5 ± 32.1^∧∧∧^	238.7 ± 87.4^###^	< 0.001
HbA_1c_ (%)	5.3 ± 0.3	5.9 ± 0.2^∧∧∧^	6.8 ± 1.9^###^	< 0.001
Glycated albumin (%)	13.2 ± 1.3	14.5 ± 1.5^∧∧∧^	21.6 ± 7.2^##^	< 0.001
Fasting insulin (μU/mL)	6.2 ± 2.7	8.1 ± 4.5	10.7 ± 9.0^##^	< 0.001
Fasting C-peptide (ng/mL)	1.5 ± 0.5^*^	2.2 ± 0.7^∧^	2.6 ± 1.1^###^	< 0.001
HOMA-IR	1.37 ± 0.60	2.17 ± 1.24^∧∧∧^	4.90 ± 6.13^###^	< 0.001
HOMA-β	91.8 ± 76.3^*^	66.9 ± 39.0^∧∧^	42.2 ± 31.3^###^	< 0.001
AST (U/L)	19.5 ± 4.9	22.3 ± 9.4^∧^	27.9 ± 19.4^##^	< 0.001
ALT (U/L)	16.3 ± 6.5	24.5 ± 20.2^∧∧^	34.3 ± 26.5^###^	< 0.001
Creatinine (mg/dL)	0.76 ± 0.13	0.75 ± 0.17	0.76 ± 0.18	0.943
eGFR (ml/min/1.73m^2^)	102.3 ± 16.8^**^	91.1 ± 16.7	93.3 ± 19.8^#^	0.003
Total cholesterol (mg/dL)	190.1 ± 37.0	198.8 ± 43.2	196.3 ± 48.0	0.495
Triglycerides (mg/dL)	103.3 ± 68.7	138.6 ± 70.1^∧^	199.3 ± 215.4^##^	0.001
HDL cholesterol (mg/dL)	56.0 ± 12.7^*^	51.0 ± 11.1^∧∧^	45.1 ± 8.9^###^	< 0.001
LDL cholesterol (mg/dL)	114.0 ± 35.2	120.8 ± 39.3	112.7 ± 39.2	0.374
CRP (mg/L)	0.43 ± 0.29	1.02 ± 0.91	1.15 ± 0.86^#^	< 0.001

Data are expressed as mean ± standard deviation of the mean or n (%).

* P < 0.05;

** P < 0.01;

*** P < 0.001 between normal and IFG.

∧ P < 0.05;

∧∧ P < 0.01;

∧∧∧ P < 0.001 between IFG and type 2 diabetes.

# P < 0.05;

## P < 0.01;

### P < 0.001 between normal and type 2 diabetes.

§ Levels of postprandial glucose were measured in 17.1% (n = 7), 68.2% (n = 58), and 88.0% (n = 81) in individuals with normal, IFG, and type 2 diabetes, respectively.

BMI, body mass index; CRP, C-reactive protein; DBP, diastolic blood pressure; eGFR, estimated glomerular filtration rate; HDL, high density lipoprotein; HOMA-β, Homeostasis model assessment of pancreatic beta cell; HOMA-IR, Homeostasis model assessment of insulin resistance; IFG, impaired fasting glucose; LDL, low density lipoprotein; SBP, systolic blood pressure.

### Serum netrin-1 levels in subjects with normal, IFG, or type 2 diabetes

**Figure 1:**
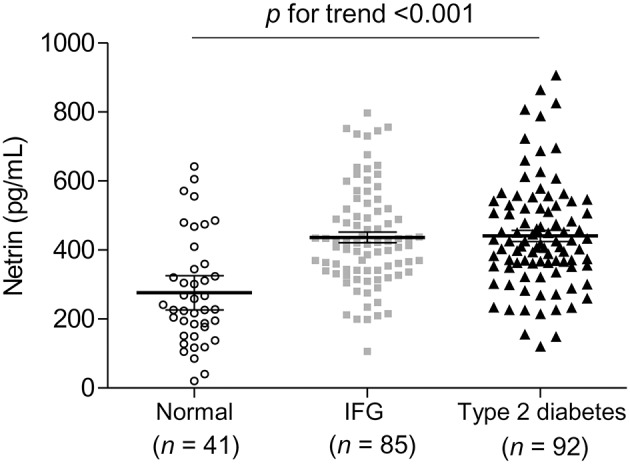
Comparison of serum netrin concentrations according to glycemic status. Each horizontal line indicates the mean with 95% confidence interval. IFG, impaired fasting glucose.

**Figure 2:**
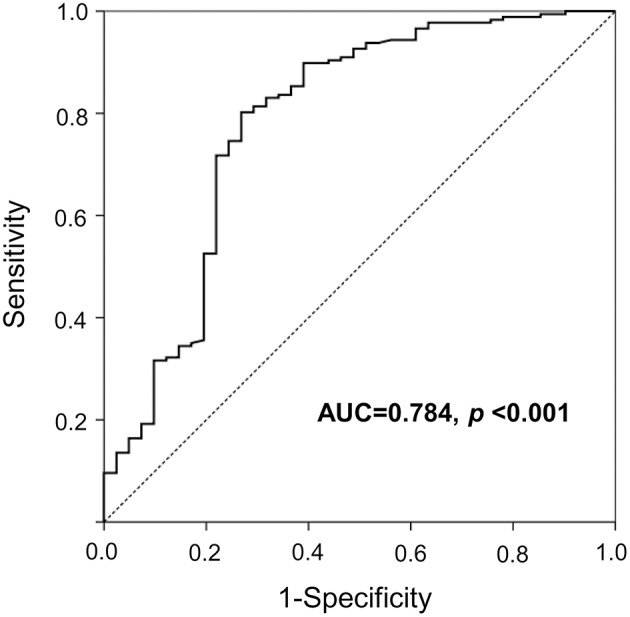
A ROC curve with serum netrin concentrations for the prediction of IFG or type 2 diabetes. IFG, impaired fasting glucose; ROC, receiver operating characteristic.

As shown in Figure 1, compared to normal controls mean serum netrin-1 levels were significantly higher in subjects with type 2 diabetes or IFG (441.0 pg/mL, 436.6 pg/mL and 275.9 pg/mL, respectively; *P* for trend < 0.001). Furthermore, Figure 2 shows the ROC curve of serum netrin-1 levels to predict IFG or type 2 diabetes. The AUC was 0.784 (95% CI = 0.693–0.875; *P* < 0.001). The use of a cutoff value of 324.9 pg/mL for serum netrin-1 was associated with the highest value to predict IFG or type 2 diabetes with a sensitivity and specificity of 80.2% and 73.2%, respectively.

### Relationship between serum netrin-1 levels and glycometabolic parameters

To evaluate the association between serum netrin-1 levels and various clinical and glycometabolic parameters, Spearman correlation analysis was performed (Table 2). Serum netrin-1 levels had strongly positive correlations with age (*r* = 0.473, *P* < 0.001), male gender (*r* = 0.213, *P* = 0.002), and statin use (*r* = 0.202, *P* = 0.003). HbA_1c_ (*r* = 0.292, *P* < 0.001), fasting glucose (*r* = 0.243, *P* < 0.001), insulin (*r* = 0.149, *P* = 0.049), C-peptide (*r* = 0.258, *P* = 0.002), HOMA-IR (*r* = 0.214, *P* = 0.004), AST (*r* = 0.223, *P* = 0.001), and ALT (*r* = 0.142, *P* = 0.038) values were also significantly associated with serum netrin-1 levels. Meanwhile, statistically inverse correlations were found between netrin-1 and HDL cholesterol (*r* = −0.229, *P* = 0.001) and eGFR (*r* = −0.468, *P* < 0.001). Multivariable-adjusted regression analyses were conducted to investigate the independent association between serum netrin-1 level and IFG or type 2 diabetes (Table 3). After adjusting for sex, age, BMI, fasting plasma glucose, HbA_1c_, ALT, HDL cholesterol, eGFR, and statin use, IFG or type 2 diabetes was independently associated with serum netrin-1 levels (standardized [STD] β = 0.405, *P* < 0.001; Model 2). The significant independent association persisted after further adjustment for HOMA-IR (STD β = 0.425, *P* = 0.008; Model 3).

**Table 2:** Correlation between serum netrin-1 concentration and glycometabolic parameters.

**Variables**	**R**	***P*-value**
Age (years)	0.473	< 0.001
Female sex	−0.213	0.002
Current alcohol drinker	0.118	0.083
Current smoker	−0.018	0.792
SBP (mmHg)	0.039	0.611
DBP (mmHg)	0.018	0.809
BMI (kg/m^2^)	0.125	0.078
Statin use	0.202	0.003
Fasting glucose (mg/dL)	0.243	< 0.001
2-h postprandial glucose (mg/dL)	0.046	0.579
HbA_1c_ (%)	0.292	< 0.001
Glycated albumin (%)	0.004	0.970
Fasting insulin (μU/mL)	0.149	0.049
Fasting C-peptide (ng/mL)	0.258	0.002
HOMA-IR	0.214	0.004
HOMA-β	−0.113	0.135
AST (U/L)	0.223	0.001
ALT (U/L)	0.142	0.038
Creatinine (mg/dL)	0.256	< 0.001
eGFR (ml/min/1.73m^2^)	−0.468	< 0.001
Total cholesterol (mg/dL)	−0.009	0.896
Triglycerides (mg/dL)	0.128	0.065
HDL cholesterol (mg/dL)	−0.229	0.001
LDL cholesterol (mg/dL)	0.074	0.286
CRP (mg/L)	0.234	0.059

*BMI, body mass index; CRP, C-reactive protein; DBP, diastolic blood pressure; eGFR, estimated glomerular filtration rate; HDL, high density lipoprotein; HOMA-β, Homeostasis model assessment of pancreatic beta cell; HOMA-IR, Homeostasis model assessment of insulin resistance; LDL, low density lipoprotein; SBP, systolic blood pressure*.

**Table 3:** Multivariable-adjusted regression analysis of the correlation between serum netrin-1 levels and IFG or type 2 diabetes (*n* = 218).

	**Model 1**		**Model 2**		**Model 3**
	*R*^2^	0.326	*R*^2^	0.405	*R*^2^	0.425
	STD β	*P-*value	STD β	*P-*value	STD β	*P-*value
IFG or Type 2 Diabetes	0.266	< 0.001	0.293	< 0.001	0.251	0.008
Sex	−0.209	0.001	−1.654	0.100	−0.136	0.114
Age	0.394	< 0.001	0.264	0.002	0.347	0.001
BMI	−0.028	0.654	0.005	0.949	−0.044	0.629
HbA_1c_	–	–	−0.006	0.938	−0.055	0.523
ALT	–	–	−0.028	0.715	−0.079	0.351
HDL cholesterol	–	–	−0.149	0.034	−0.103	0.176
statin user	–	–	0.061	0.338	0.032	0.642
eGFR	–	–	−0.165	0.063	−0.113	0.261
HOMA-IR	–	–	–	–	0.223	0.026

Log-transformation was used for variables (HbA_1c_, ALT, HDL cholesterol, eGFR, and HOMA-IR).

*ALT, alanine aminotransferase; BMI, body mass index; eGFR, estimated glomerular filtration rate; HDL, high density lipoprotein; HOMA-IR, Homeostasis model assessment of insulin resistance; LDL, low density lipoprotein; STD, standardized*.

## Discussion

The present study demonstrates, for the first time, that serum netrin-1 level may serve as a sensitive and early indicator of the development of IFG and type 2 diabetes. Serum netrin-1 concentrations were significantly higher in individuals with IFG or type 2 diabetes, and serum netrin-1 level was significantly positively correlated with fasting glucose, HbA_1c_, HOMA-IR, AST, and ALT. Serum netrin-1 was also independently associated with IFG or type 2 diabetes after adjusting for covariates and potential confounders. Moreover, the AUC to predict IFG and type 2 diabetes was 0.784.

Netrin-1 belongs to the laminin-related proteins of axon-guidance and has been reported to play diverse roles through its two classic receptor families, such as deleted in colorectal cancer (DCC) subfamily (e.g., DCC and Neogenin) and uncoordinated 5 (UNC5) subfamily (e.g., UNC5A-UNC5D), expressed by the target cells (8). Anti-inflammatory actions of netrin-1 were reported as inhibition of migration of leukocytes and protection from vascular inflammation, peritonitis, and pancreatitis through UNC5B receptor (10, 17, 31). Urinary netrin-1 was elevated in patients with acute kidney injury after cardiac surgery and was suggested to be an early predictive biomarker of acute kidney injury before the rise of serum creatinine (32). In addition, urinary netrin-1 was elevated early in the time course of rat and human diabetes (33, 34). However, the purinergic signaling events by netrin-1 during renal damage demonstrated a protective role of endogenous netrin-1 in ameliorating disease activity (35). Overexpression of netrin-1 in kidney proximal tubule suppressed inflammation and albuminuria through suppression of diabetes-induced cyclooxygenase-2 (COX-2) expression and prostaglandin E2 (PGE2) production by inhibiting nuclear factor kappa B (NF-kB) activation (36). Netrin-1 suppressed Th1/Th2/Th17 cytokines (e.g., IL-6, IFN-γ, and TNF-α) and reduced inflammation of renal perfusion injury through UNC5B receptor (37). Recent studies revealed that netrin-1 interacted with the adenosine 2B receptor (A2BAR) expressed on polymorphonuclear neutrophils (PMNs) to control local inflammation during hypoxia and promoted liver repair and regeneration (14, 16, 38).

Previously, Ay and colleagues reported that serum netrin-1 levels were raised in patients with diabetes whose mean HbA_1c_ level was 8.1% compared to non-diabetic patients (39). Also, there was a strong positive correlation between plasma netrin-1 level and HbA_1c_, and a significant negative correlation between eGFR, but the study sample was relatively small and a comparative group (such as pre-diabetes) was not included. In the present study, serum netrin-1 was significantly elevated in individuals with IFG or type 2 diabetes compared to normal controls, which is probably attributable to a compensatory response to IFG or type 2 diabetes. Although mean HbA_1c_ values were 6.8% in newly-diagnosed drug-naïve patients with type 2 diabetes and 5.9% in individuals with IFG, we found grade-dependent levels of serum netrin-1 according to group, suggesting netrin-1 may be a sensitive indicator for IFG or early type 2 diabetes. In addition, serum netrin-1 levels were significantly positively correlated with fasting glucose, HbA_1c_, and HOMA-IR whereas they showed a significant negative correlation with eGFR and HDL cholesterol. In a previous report, obese individuals (mean BMI 38.6 kg/m^2^) showed lower circulating netrin-1 than lean (mean BMI 18.2 kg/m^2^), whereas, serum netrin-1 levels were similar in HFD-fed and chow-fed mice (40). However, BMI was not significantly related with levels of serum netrin-1 in our study (mean BMI 25.2 kg/m^2^) and another study (mean BMI about 24 kg/m^2^) (41). Liu and colleagues showed that in patients with newly diagnosed type 2 diabetes, netrin-1 levels were significantly lower than in normal controls. There was a negative association between levels of circulating netrin-1 and fasting and postprandial blood glucose, HbA1c, triglycerides, and HOMA-IR which were different from our results (41). These might be explained by distinct characteristics of study population. We measured circulating levels of netrin-1 and other laboratory parameters at the time of diagnosis of type 2 diabetes, showing mean values of HbA1c 6.8% and HOMA-IR 4.90. However, Lui et al. recruited patients who had been diagnosed with type 2 diabetes within 6 months, having mean values of HbA1c 8.5% and HOMA-IR 1.13, indicating more hyperglycemic but not insulin resistant phenotypes. Furthermore, different commercial netrin-1 ELISA kits can affect this inconsistent finding. In the previous report, mean circulating levels of netrin-1 were 1.77 pg/mL in normal controls and 0.96 pg/mL in patients with type 2 diabetes, but 275.9 pg/mL in normal controls and 441.0 pg/mL in patients with type 2 diabetes in our study. Similar to our findings, other reports observed that mean values of serum netrin-1 levels were about 490 pg/mL in obese individuals (40, 42). In diabetic nephropathy mice model, the mean level of serum netrin-1 was about 100 pg/mL (35). Different conditions of study populations, sampling, ELISA kits, and techniques for measuring netrin-1 may contribute to the discrepancy in results and these should be considered when interpreting the data. Further research with a larger sample size may be needed to improve replicability. The role of netrin-1 is also implicated in pancreatic morphogenesis, tissue remodeling, and migration of pancreatic epithelial cells, including duct-cell and fetal islet cell (43, 44). Recently, Gao et al. showed a direct stimulatory effect of netrin-1 on insulin secretion in isolated mouse islets by promoting beta-cell calcium ion influx, and cyclic adenosine 5'-monophosphate (cAMP) production (45). In the previous study, improvement of beta-cell function, demonstrated as increased islet insulin content and plasma insulin levels, normalized plasma glucagon levels, and enhanced islet vascularization, were shown in high fat diet/streptozotocin-induced diabetic mice after netrin-1 administration (45). In addition, netrin-1-treated diabetic mice presented a substantial reduction in macrophage infiltration in pancreatic islets and a decrease in circulating TNF-α levels, showing the anti-inflammatory action of netrin-1 in diabetes. In diabetic mice, expression of netrin-1 was decreased in the aorta but, overexpression of netrin-1 prevented from diabetes-induced vascular damage and attenuated high glucose-induced oxidative stress (46). However, there is no longitudinal study of changes in netrin-1 levels during the development of diabetes and insulin resistance, and the role of netrin-1 in the pathophysiology of type 2 diabetes is unclear. Taken together, a possible beneficial compensatory response of netrin-1 to the changes that happen early in the time course of diabetes needs to be studied. This perspective of netrin-1 as a modulator of inflammatory response and regeneration in pancreas, endogenous circulating netrin-1 may be implicated in the pathophysiologic mechanism of IFG or type 2 diabetes (25). In contrast, controversies from different aspects of netrin-1 applied to various sites. High fat diet-fed obese mice showed higher expression of netrin-1 and UNC5B mRNA and macrophage retention in visceral adipose tissue compared to lean chow-fed mice, and deletion of hematopoietic netrin-1 facilitated adipose tissue macrophage emigration, reduced inflammation, and improved insulin resistance in obese mice (40).

There are several distinguishing aspects of this study. First, we included drug-naïve, newly-diagnosed patients with type 2 diabetes, minimizing any effect of anti-diabetes medication on serum netrin-1 concentrations. Second, we identified serum netrin-1 levels as a possible predictor of both IFG and type 2 diabetes. When we compared netrin-1 levels according to glycemic status, we found that values were higher in individuals with IFG and highest in patients with type 2 diabetes compared with healthy controls. As a potential predictive indicator for IFG or type 2 diabetes, netrin-1 has the substantial clinical potential to prevent diabetes or slow its progression. Third, our sample sizes were larger than in previous reports, increasing the reliability of results (33, 39–41). Finally, we conducted multivariable-adjusted regression analyses to investigate the independent relationships between netrin-1 level and IFG or type 2 diabetes adjusting for potential confounders, including eGFR and HOMA-IR.

Notwithstanding, this study has several limitations. Due to the cross-sectional design of the study, we cannot infer a causal relationship between an increased netrin-1 concentration and IFG or early stage of type 2 diabetes. In addition, we could not measure levels of albuminuria at enrollment, but levels of eGFR to determine renal function, which was included in the analyses as a covariate. As levels of postprandial glucose levels were not measured from all the participants, the diagnosis of normal controls and subjects with IFG could be affected and overestimated. Also, various inflammatory cytokines were not included in the analyses. Moreover, investigation of the mechanism of action between netrin-1 level and pathogenesis of IFG and early stage of type 2 diabetes is needed to further our understanding.

## Conclusion

In conclusion, we report that elevated serum netrin-1 levels are significantly associated with IFG or newly diagnosed type 2 diabetes. Further prospective studies are needed to elucidate the role of netrin-1 in the pathogenesis of type 2 diabetes and expand its potential for diagnosis and treatment.

## Data availability

The datasets used and/or analyzed during this study are available from the corresponding author on reasonable request.

## Author contributions

JY wrote the manuscript and researched data. GK wrote the manuscript and contributed to statistical analysis. B-WL and B-SC researched data and reviewed the manuscript. EK, J-HK, and JC reviewed the manuscript and contributed to the discussion. S-GL designed the study and reviewed the manuscript. YL recruited patients and contributed to the study design and discussion.

### Conflict of interest statement

The authors declare that the research was conducted in the absence of any commercial or financial relationships that could be construed as a potential conflict of interest.

## Abbreviations

A2BAR: adenosine 2B receptor
ALT: alanine aminotransferase
AST: aspartate aminotransferase
AUC: areas under the receiver operating characteristic curves
CKD-EPI: Chronic Kidney Disease Epidemiology Collaboration
COX-2: cyclooxygenase-2
DCC: deleted in colorectal cancer
IFG: impaired fasting glucose
OD: optical density
PGE2: prostaglandin E2
PMNs: polymorphonuclear neutrophils
ROC: receiver operating characteristic
STD: standardized
UNC5: uncoordinated 5
VIF: variance inflation factor.
