# Supplementary material for: Relationship Between Circulating Netrin-1 Concentration, Impaired Fasting Glucose, and Newly Diagnosed Type 2 Diabetes
Source: Front Endocrinol (Lausanne). 2018 Nov 23;9:691. doi: 10.3389/fendo.2018.00691 (PMC6265472; doi:10.3389/fendo.2018.00691)
Supplement: Supplementary file 1 [file Table_1.DOCX]

**Supplement data for ELISA kit data**

Each ELISA kit included sera from all the three categories of individuals examined as following Table.

|  | ELISA kit 1 | | ELISA kit 2 | | ELISA kit 3 | | ANOVA* |
| --- | --- | --- | --- | --- | --- | --- | --- |
|  | Number | Mean | Number | Mean | Number | Mean | p-value |
| Subjects with IFG | 22 | 408.4 | 15 | 462.4 | 48 | 441.5 | 0.514 |
| Subjects with T2DM | 32 | 400.2 | 58 | 466.4 | 2 | 360.1 | 0.118 |
| Normal controls | 27 | 263.2 | 11 | 241.3 | 3 | 517.5 | 0.015 |
| Total | 81 |  | 84 |  | 53 |  |  |

* The difference in mean netrin-1 concentration among ELISA kits in each category was analyzed by ANOVA.

The table showed the number of subject and mean Netrin-1 concentration in each category according to the ELISA kit. The mean value of subjects with IFG was not different among ELISA kit (p-value, 0.514). Similarly, the mean value of subjects with newly diagnosed type 2 diabetes was not significantly different among ELISA kit (p-value, 0.118). Although the mean value of normal controls in ELISA kit 3 was significantly higher than other ELISA kits, the number of normal control included in ELISA kit 3 was very small (only 3 subjects). Therefore, this difference might be caused by subject specific extreme value rather than by ELISA kit.

Although there was no internal control materials, all tested ELISA kits were same lot number and the assays were performed by one researcher on same day. A total of five calibrators were used except blank, and the blank optical density (OD) value was subtracted from each OD value, and then concentration was calculated by calibration curve.
